# Impact of skeletal muscle mass in patients with unresectable gastric cancer who received palliative first-line chemotherapy based on 5-fluorouracil

**DOI:** 10.1186/s12885-021-08953-8

**Published:** 2021-11-13

**Authors:** Tomoyuki Matsunaga, Hiroaki Saito, Wataru Miyauchi, Yuji Shishido, Kozo Miyatani, Masaki Morimoto, Yuki Murakami, Takehiko Hanaki, Kyoichi Kihara, Manabu Yamamoto, Naruo Tokuyasu, Shuichi Takano, Teruhisa Sakamoto, Toshimichi Hasegawa, Yoshiyuki Fujiwara

**Affiliations:** 1 Department of Surgery, Division of Gastrointestinal and Pediatric Surgery, School of Medicine, Tottori University Faculty of Medicine, 36-1 Nishi-cho, Yonago, 683-8504 Japan; 2 Department of Surgery, Japanese Red Cross Tottori Hospital, 117 Shotoku-cho, Tottori, 680-8517 Japan

**Keywords:** Gastric cancer, Chemotherapy, Skeletal muscle mass

## Abstract

**Background:**

The mortality rate of patients with unresectable gastric cancer (UGC) has decreased with the development of chemotherapies and surgical techniques. However, the survival rate remains low. We retrospectively examined the prognostic significance of the pretreatment skeletal muscle mass index (SMI) and nutritional and inflammatory factors in patients with UGC.

**Methods:**

This study included 83 patients diagnosed with UGC at Tottori University Hospital who received palliative chemotherapy based on 5-fluorouracil. Pretreatment computed tomography (CT) measured overall skeletal muscle mass (SMM) and cross-sectional SMM at the third lumbar vertebra (L3). We focused on the neutrophil-to-lymphocyte ratio (NLR), C-reactive protein-to-albumin ratio (CAR), prognostic nutritional index (PNI), and platelet-to-lymphocyte ratio (PLR) as nutritional and inflammatory factors.

**Results:**

Receiver operating characteristic curve analysis was performed for median survival time (MST) after palliative chemotherapy. SMIs for males and females (43.9 cm^2^/m^2^ and 34.7 cm^2^/m^2^, respectively) were the cutoff values, and patients were divided into high (SMI^High^; *n* = 41) and low SMI groups (SMI^Low^; *n* = 42). Body mass index (BMI) was significantly higher in patients in the SMI^High^ group than in the SMI^Low^ group (*p* < 0.001). The number of patients who received third-line chemotherapy was significantly higher in the SMI^High^ group than in the SMI^Low^ group (*p* = 0.037). The MST was significantly higher in the SMI^High^ group than in the SMI^Low^ group (17.3 vs. 13.8 months; *p* = 0.008). The incidence of grade 3 or 4 side effects was significantly higher in patients with SMI^Low^ UGC (*p* = 0.028). NLR was significantly higher in patients with SMI^Low^ than it was in those with SMI^High^. (*p* = 0.047). In the univariate analysis, performance status, SMI, histological type, lines of chemotherapy, and NLR were prognostic indicators. The multivariate analysis identified SMI (*p* = 0.037), NLR (*p* = 0.002), and lines of chemotherapy (*p* < 0.001) as independent prognostic factors.

**Conclusions:**

The SMI^Low^ group had significantly more grade 3 or 4 side effects, were related to high NLR, and had a significantly worse prognosis than the SMI^High^ group.

**Trial registration:**

Retrospectively registerd.

## Introduction

Gastric cancer is the fifth most common and third leading cause of cancer in the world [1]. The main strategy for gastric cancer is gastrectomy. However, the prognosis is poor because recurrence is common after gastrectomy, and patients with gastric cancer are often diagnosed with metastasis to other organs [2, 3]. The main strategy for unresectable gastric cancer (UGC) is chemotherapy. The mortality rate decreases with the development of chemotherapies and surgical techniques; however, the survival rate remains low [4, 5]. Many combination chemotherapy regimens have been studied in randomized trials. Five-fluorouracil (5-FU) is a key drug used in combination with other drugs for first-line chemotherapy in patients with UGC [6–8].

Sarcopenia is a disease defined by loss of skeletal muscle mass (SMM) and function. Its prognosis has been reported to be poor in various cancers, including gastric cancer [9, 10]. In advanced gastric cancer with metastasis to another organ, most patients suffer from poor dietary intake, resulting in inadequate nutrition. In patients with recurrent gastric cancer, gastrectomy reduces the stomach’s capacity to digest, decreasing meal intake and resulting in weight and SMM loss. These findings suggest that patients with UGC already have reduced SMM at the time of diagnosis. Sarcopenia may influence chemotherapy pharmacokinetics, which could be associated with the adverse effects of chemotherapy in several cancers [11, 12]. However, there are no reports of an association between SMM and side effects or prognosis in patients with UGC treated with 5-FU based chemotherapy. Furthermore, the reason for the relationship between sarcopenia and poor cancer prognosis remains unclear. On the other hand, nutritional status or inflammation could be a prognostic factor for patients with cancer.

Recently, the relationship between nutrition-based and inflammation-based markers and prognosis has also been reported in various cancers, including gastric cancer [13, 14]. Many inflammatory markers that can be used to predict prognosis have been reported, such as the C-reactive protein (CRP)-to-albumin ratio (CAR), neutrophil-to-lymphocyte ratio (NLR), platelet-to-lymphocyte ratio (PLR), and the prognostic nutritional index (PNI). However, the relationship between sarcopenia and inflammatory markers is still unclear.

This study investigated the relationship between SMM, chemotherapy side effects, and prognosis in patients with UGC. We also investigated the relationship between SMM and nutritional and inflammatory markers.

## Patients and methods

### Patients

Between January 2008 and December 2019, 47 patients were pathologically diagnosed with unresectable advanced gastric cancer, and 67 patients developed recurrence after undergoing curative gastrectomy for gastric cancer at Tottori University Hospital. Of those 114 patients, 83 received palliative first-line chemotherapy based on 5-fluorouracil (5-FU) and were analyzed in this study. The Japanese gastric cancer treatment guidelines were used to determine clinicopathological findings [7]. Clinical data, such as age, sex, Eastern Cooperative Oncology Group Performance Status (ECOG PS), and type of metastatic site at the time of diagnosis of unresectable advanced gastric cancer or recurrence, were collected from electronic medical records. The follow-up schedule of patients who underwent curative gastrectomy was every three months to check for recurrence by performing blood tests and physical examination. After the operation, CT was performed at least every six months. The causes of death were examined from clinical records. CT and positron emission tomography CT were used to detect unresectable lesion or recurrence patterns. Overall survival (OS) was defined as the time from initiation of first-line chemotherapy until death from any cause or the last follow-up.

### Definition of skeletal muscle mass index

**Fig. 1:**
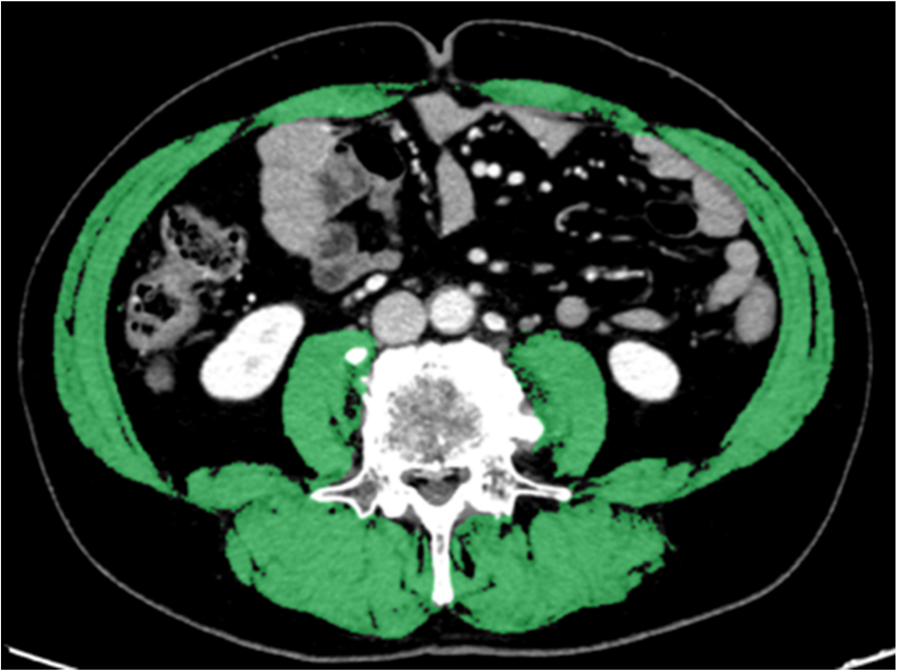
Measurement of skeletal muscle mass in patients with unresectable gastric cancer. Axial computed tomography slice of the third lumbar vertebra. Green areas indicate skeletal muscle mass. Abbreviations: SMM, skeletal muscle mass; UGC, unresectable gastric cancer

All patients were diagnosed as having UGC by CT, and the CT was used as pretreatment CT to measure SMM. All patients received first-line chemotherapy within 3 weeks of receiving the pretreatment CT. Pretreatment CT was performed to measure SMM, and a three-dimensional medical image analysis system (SYNAPSE VINCENT, FujiFilm Corporation, Tokyo, Japan) was used to measure the cross-sectional SMM at the level of L3 [15]. The areas covered by SMM were calculated from pixels in the density range of − 29 to + 150 Hounsfield Units, which included muscle and intra-abdominal organs but excluded bone and fat [16]. The L3 region comprised the psoas, paraspinal, and abdominal wall muscles (Fig. 1). The skeletal muscle area in a single abdominal image was proportional to the whole-body muscle mass [17]. SMI was defined as the muscle area normalized by the square of the height (m^2^) [18].

### Details of first-line chemotherapy

The standard first-line palliative systemic chemotherapy was based on the 5-FU regimen administered per the gastric cancer treatment guidelines for each decade [19, 20]. At the physician’s discretion, a single agent was used in patients with an ECOG PS of ≥2, those older than 80 years, or those who refused combined chemotherapy. Monotherapy was administered in 13 patients, and combination chemotherapy was administered in 70 patients in this study. The monotherapy regimen was the S-1 regimen, and the combination chemotherapy regimens were as follows: (1) combined S-1 + cisplatin (*n* = 19), (2) combined S-1 + paclitaxel + intraperitoneally infused paclitaxel (*n* = 15), (3) combined S-1 + oxaliplatin (*n* = 13), (4) combined S-1 + docetaxel (*n* = 10), (5) combined capecitabine + oxaliplatin (*n* = 5), (6) combined capecitabine + cisplatin (*n* = 3), (7) combined capecitabine + trastuzumab (n = 3), and (8) combined S-1 + cisplatin + docetaxel (*n* = 2). Of the 83 patients who underwent chemotherapy for UGC, 67 patients (80.7%) received second-line chemotherapy, and 41 patients (49.4%) received third-line chemotherapy.

### Definition of side effects

The National Cancer Institute Common Toxicity Criteria version 4.0 was used to grade the chemotherapy side effects [21]. We examined the side effects observed in the first three cycles of first-line chemotherapy. A higher grade was used in patients with multiple side effects. We focused on hematologic toxicity, febrile neutropenia (FN), and gastrointestinal toxicity.

### Definition of nutrition-based and inflammation-based factors

The nutrition- and inflammation-based prognostic scores in this study were the CAR, which was the CRP level divided by the albumin level (CRP measured in mg/L, and albumin measured in g/L) [22]; the NLR [23]; the PNI, which was calculated by the formula 10 × albumin (g/dL) + 0.005 × lymphocyte count/μL [24]; and the PLR [13]. All indicators involved in calculating nutrition- and inflammation-based prognostic scores were measured within seven days before chemotherapy. All measurements were recorded from the same blood sample.

### Statistical analysis

The Mann–Whitney U test was used to compare continuous variables. Categorical variables were compared by the Fisher’s exact test or χ^2^ test. The Kaplan–Meier method was used to calculate survival curves, and differences between survival curves were examined using the log-rank test. The Cox’s proportional hazards model was used to perform the univariate and multivariate analyses of OS prognostic factors. *P* < 0.05 was considered significant. SPSS software (SPSS for Windows version 24; IBM Corp., Armonk, NY, USA) was used for all statistical analyses.

## Results

### Patient characteristics and prognosis in patients with UGC

Table 1 summarizes the characteristics of patients included in the current study. Overall, there were 61 (73.4%) males and 22 (26.6%) females, and their average age was 65.4 ± 12.4 years (range 32–84). The ECOG PS status of 41, 38, and 4 patients was 0, 1, and 2, respectively. The average SMI was 42.8 ± 7.7 cm^2^/m^2^, and the average BMI was 20.0 ± 3.8 cm^2^/m^2^. The cause of the unresectable condition was advanced disease in 39 cases and recurrent disease in 44 cases. The most common metastatic site was the peritoneum, followed by lymph nodes and hematogenous metastasis. Regarding histology, 38 patients had a differentiated-type carcinoma, and 45 had an undifferentiated-type carcinoma.

**Table 1:** Clinicopathological characteristics of patients with SMI^High^ and SMI^Low^ UGC

	All patients (*n* = 83)	SMI^High^ (*n* = 41)	SMI^Low^ (*n* = 42)	*p* value
Age (years)	65.4 ± 12.4	64.9 ± 14.5	67.9 ± 9.2	0.176
Sex				0.119
Male	61 (73.5)	27 (65.9)	34 (81.0)	
Female	22 (26.5)	14 (34.1)	8 (19.0)	
ECOG PS (0/1/2)				0.209
0	41 (49.4)	24 (58.5)	17 (40.5)	
1	38 (45.8)	16 (39.0)	22 (52.4)	
2	4 (4.8)	1 (2.5)	3 (7.1)	
BMI	20.0 ± 3.8	21.5 ± 4.3	18.6 ± 2.5	< 0.001
SMI	42.8 ± 7.7	47.4 ± 7.3	38.2 ± 5.0	< 0.001
Unresectable cause				0.907
Advanced cases	39 (47.0)	19 (46.3)	20 (47.6)	
Recurrent cases	44 (53.0)	22 (53.7)	22 (52.4)	
Histologic type				0.222
Differentiated	38 (45.8)	16 (39.0)	22 (52.4)	
Undifferentiated	45 (54.2)	25 (61.0)	20 (47.6)	
Peritoneum metastases				0.903
Positive	37 (44.6)	18 (43.9)	19 (45.2)	
Negative	46 (55.4)	23 (56.1)	23 (54.8)	
Lymph node metastases				0.898
Positive	35 (42.2)	17 (41.5)	18 (42.9)	
Negative	48 (57.8)	24 (58.5)	24 (57.1)	
Hematogenous metastases				0.867
Positive	25 (30.1)	12 (29.3)	13 (31.0)	
Negative	58 (69.9)	29 (70.7)	29 (69.0)	
Lines of chemotherapy				0.289
< 2nd line	16 (19.3)	6 (14.6)	10 (23.8)	
≧2nd line	67 (80.7)	35 (85.4)	32 (76.2)	
Lines of chemotherapy				
< 3rd line	42 (50.6)	16 (39.0)	26 (61.9)	0.037
≧3rd line	41 (49.4)	25 (61.0)	16 (38.1)	

Data are presented as mean ± standard deviation or number (percentage) of patients

BMI, body mass index; ECOG PS, Eastern Cooperative Oncology Group performance status; SMI, skeletal muscle mass; SMI^High^, high skeletal muscle mass; SMI^Low^, low skeletal muscle mass; UGC, unresectable gastric cancer

**Fig. 2:**
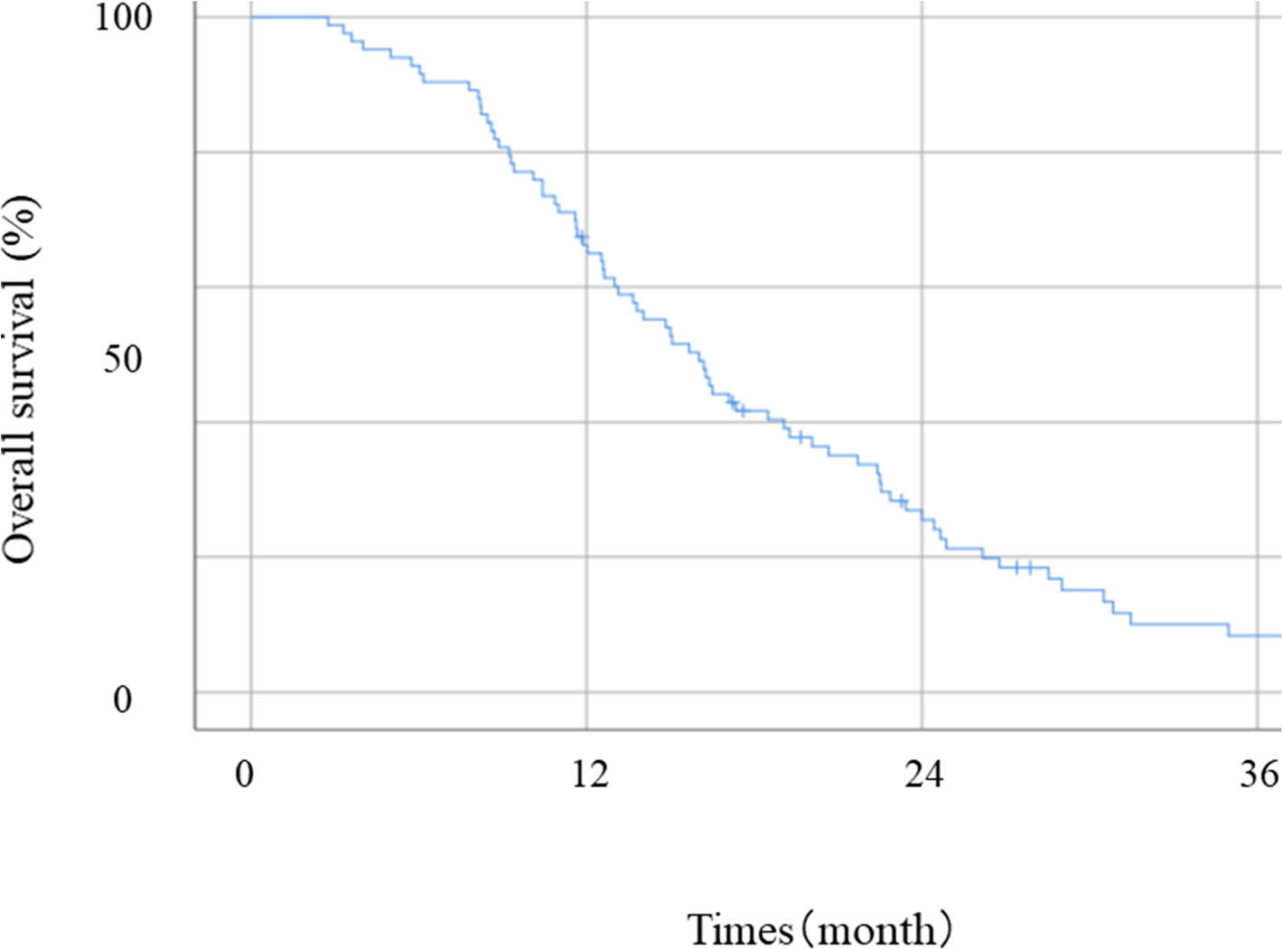
Overall survival curves in patients with unresectable gastric cancer. Abbreviations: UGC, unresectable gastric cancer

**Fig. 3:**
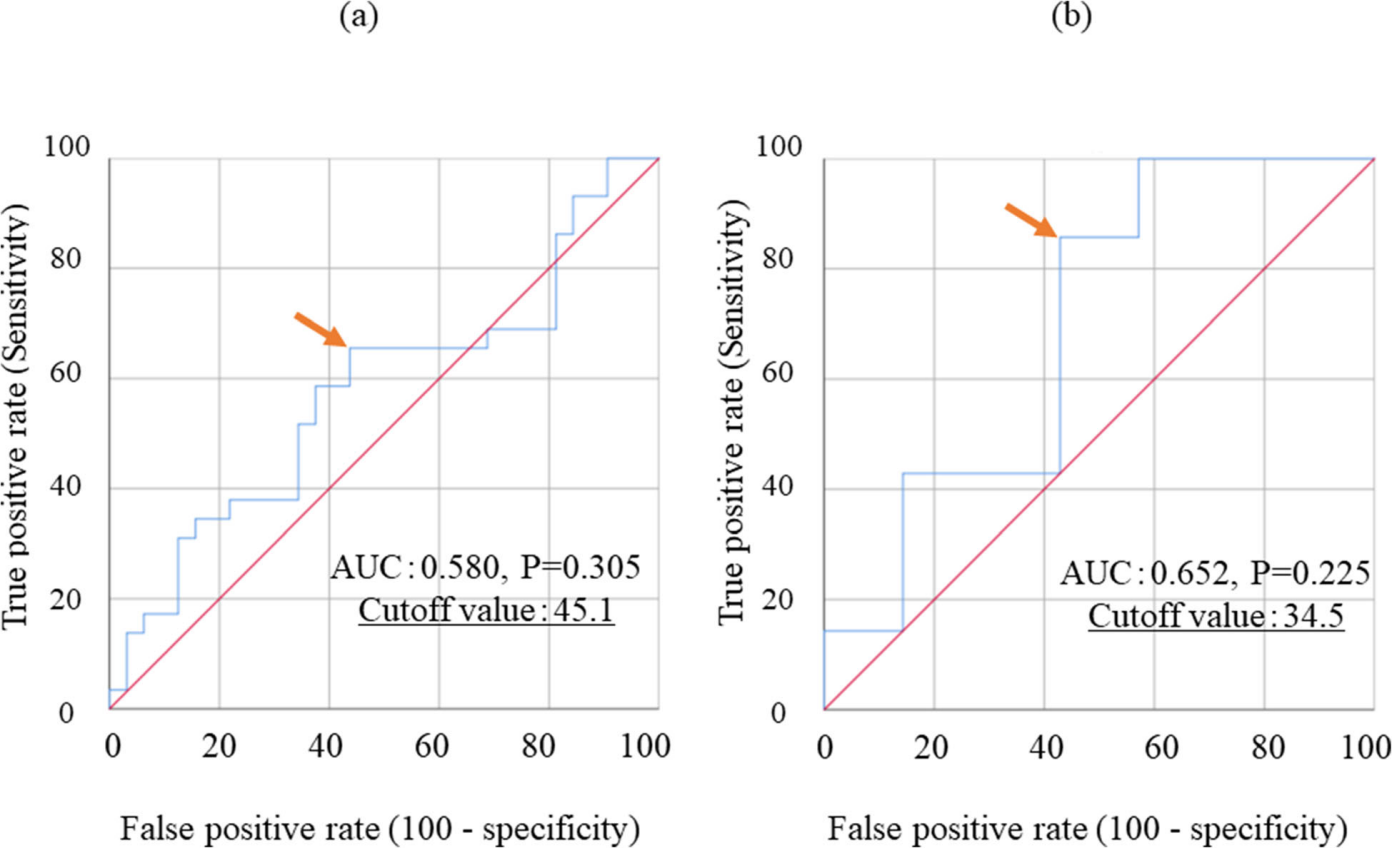
ROC curves of the SMI for the MST for males (a) and females (b). The arrow indicates the optimal cutoff value. Abbreviations: AUC, area under the curve; MST, median survival time; ROC, receiver operating characteristic; SMI, skeletal muscle index

**Fig. 4:**
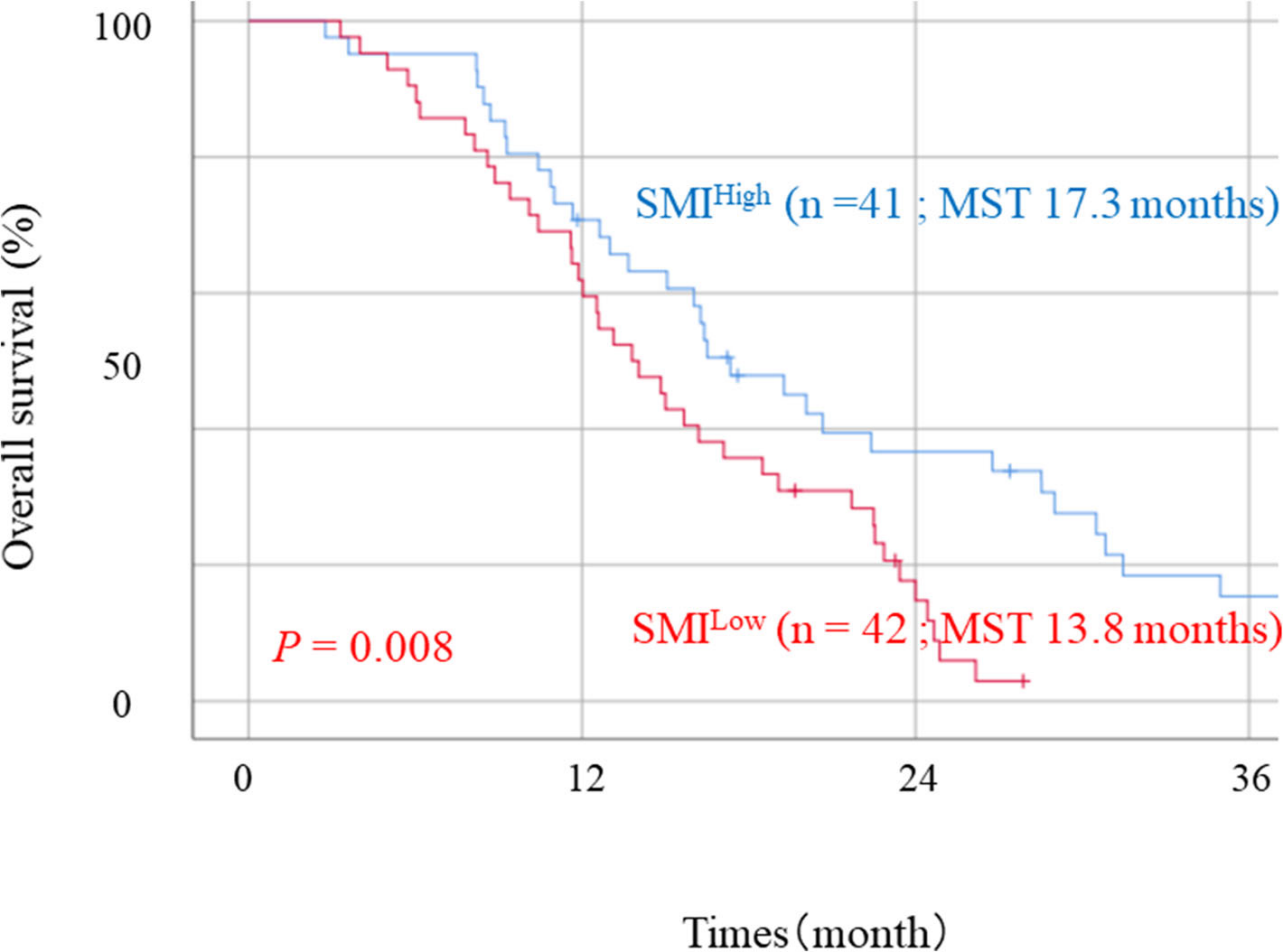
Overall survival curves according to skeletal muscle mass index in patients with unresectable gastric cancer. Abbreviations: MST, median survival time; SMI^High^, high skeletal muscle mass index; SMI^Low^, low skeletal muscle mass index; UGC, unresectable gastric cancer

The median survival rate (MST) was 16.0 months in patients with UGC (Fig. 2). The receiver operating curve analysis for the 16.0 months OS for each sex indicated that the optimal cutoff of the SMI was 45.1 cm^2^/m^2^ (males) and 34.5 cm^2^/m^2^ (females) (Fig. 3). Based on the optimal cutoff, patients were divided into a high SMI group (SMI^High^ group; *n* = 41) and a low SMI group (SMI^Low^ group; *n* = 42). Table 1 shows the relationships between SMI and clinicopathological variables of the patients. BMI was significantly higher in patients in the SMI^High^ group than in the SMI^Low^ group (*P* < 0.001). The number of patients who received third-line chemotherapy was significantly higher in the SMI^High^ group than in the SMI^Low^ group (*P* = 0.037). No significant differences were observed regarding age, gender, ECOG PS, unresectable cause, histologic type, metastatic site, and the number of patients who received second-line chemotherapy. The MST was significantly higher in the SMI^High^ group (17.3 vs. 13.8 months; *P* = 0.008, Fig. 4).

### Details of first-line chemotherapy and side effects

Table 2 presents the details of SMI and first-line chemotherapy. The SMI^L^°^w^ group tended to include more patients treated with monotherapy than the SMI^High^ group (*P* = 0.144). Table 3 presents the details of the side effects of each regimen used in first-line chemotherapy, and gastrointestinal toxicities according to each symptom are also described. Table 4 presents the details of SMI and its side effects. The incidence of all side effects of grade 3 or 4 was significantly higher in the SMI^Low^ group than in the SMI^High^ group (*P* = 0.028). The incidence of grade 3 or 4 gastrointestinal toxicity was significantly higher in the SMI^Low^ group than in the SMI^High^ group (*P* = 0.039). There were no significant differences in terms of neutropenia, anemia, thrombocytopenia, and FN.

**Table 2:** First-line chemotherapy for patients with UGC with SMI^High^ and SMI^Low^

	SMI^High^ (n = 41)	SMI^Low^ (n = 42)	P value
Monotherapy			0.144
S-1	4 (9.8)	9 (21.4)	
Combined chemotherapy	37 (90.2)	33 (78.6)	
S-1 + cisplatin	6	13	
Combined S-1 + paclitaxel + intraperitoneally infused paclitaxel	9	6	
S-1 + oxaliplatin	8	5	
S-1 + docetaxel	8	2	
Capecitabine + oxaliplatin	2	3	
Capecitabine + cisplatin	1	2	
Capecitabine + trastuzumab	2	1	
S-1 + cisplatin + docetaxel	1	1	

Data are presented as number (percentage) of patients

SMI^High^, high skeletal muscle mass; SMI^Low^, low skeletal muscle mass; UGC, unresectable gastric cancer; SMI, skeletal muscle mass

**Table 3:** The details of the side effects of each regimen used in first-line chemotherapy

													Gastrointestinal toxicity																
	Neutropenia				Anemia				Thrombocytopenia				Nausia/Vomitting				Diarrea				Constipation				Mucositis				FN
	grade				grade				grade				grade				grade				grade				grade				grade
	***1***	***2***	***3***	***4***	***1***	***2***	***3***	***4***	***1***	***2***	***3***	***4***	***1***	***2***	***3***	***4***	***1***	***2***	***3***	***4***	***1***	***2***	***3***	***4***	***1***	***2***	***3***	***4***	***3***
S-1 (n = 13)	2	2	1	0	2	2	0	0	1	1	0	0	3	2	1	0	2	0	3	0	2	0	0	0	2	0	0	0	0
S-1 + cisplatin (n = 19)	5	2	2	3	4	2	0	0	4	2	0	1	5	4	2	0	4	2	0	0	4	0	0	0	1	2	0	0	2
IP (n = 15)	2	1	3	2	1	1	0	0	2	1	0	0	2	2	0	0	1	1	1	0	3	0	0	0	1	1	0	0	1
S-1 + oxaliplatin (n = 13)	4	2	1	0	3	2	1	0	1	4	0	0	4	2	0	0	2	2	1	0	2	0	0	0	1	2	0	0	0
S-1 + docetaxel (n = 10)	2	1	0	1	3	1	0	0	2	0	0	0	3	2	1	0	1	1	1	0	2	0	0	0	2	1	0	0	0
Capecitabine + oxaliplatin (n = 5)	1	1	0	1	1	0	0	0	1	0	1	0	1	1	1	0	1	0	0	0	1	0	0	0	0	1	0	0	1
Capecitabine + cisplatin (n = 3)	2	0	0	0	1	0	0	0	1	0	1	0	1	0	1	0	0	0	0	0	1	0	0	0	1	0	0	0	0
Capecitabine + trastuzumab (n = 3)	0	0	0	0	0	0	0	0	0	0	0	0	1	0	1	0	0	0	0	0	0	0	0	0	0	0	0	0	0
S-1 + cisplatin + docetaxel (n = 2)	0	1	1	0	1	0	0	0	1	0	0	0	2	0	0	0	1	1	0	0	1	0	0	0	0	1	0	0	0

Data are presented as number of patients

FN, febrile neutropenia; IP, Combined S-1 + paclitaxel + intraperitoneally infused paclitaxel

**Table 4:** The incidence of grade 3 or 4 side effects in patients with UGC with SMI^High^ and SMI^Low^

	SMI^High^ (n = 41)	SMI^Low^ (n = 42)	P value
All side effects of grade 3 or 4			0.028
Positive	10 (24.3)	20 (47.6)	
Negative	31 (75.7)	22 (52.4)	
Neutropenia of grade 3 or 4			0.421
Positive	6 (14.6)	9 (21.4)	
Negative	35 (85.4)	33 (78.6)	
Anemia of grade 3 or 4			0.309
Positive	1 (2.4)	0	
Negative	40 (97.6)	42	
Thrombocytopenia of grade 3 or 4			0.081
Positive	0	3 (7.1)	
Negative	41	39 (92.9)	
Gastrointestinal toxicity			0.039
Positive	3 (7.3)	10 (23.8)	
Negative	38 (92.7)	32 (76.2)	
FN			0.980
Positive	2 (4.9)	2 (4.8)	
Negative	39 (95.1)	40 (95.2)	

Data are presented as number (percentage) of patients

FN, febrile neutropenia; SMI^High^, high skeletal muscle mass; SMI^Low^, low skeletal muscle mass; UGC, unresectable gastric cancer; SMI, skeletal muscle mass

#### Response to first line chemotherapy

In this study, 1 (1.2%) patient achieved a complete response, 21 (25.3%) achieved a partial response, and 35 (42.2%) achieved stable disease, with the remaining 26 patients experiencing progressive disease (PD) (31.3%). The objective response rate (ORR) and disease control rate (DCR) were 26.5% (22 of 83 patients) and 68.7% (57 of 83 patients), respectively (Table 5). No significant differences were observed regarding ORR and DCR between the SMI^High^ and SMI^Low^ group.

**Table 5:** Responses to first line chemotherapy

	All patients (n = 83)	SMI^High^ (n = 41)	SMI^Low^ (n = 42)	P value
**Best overall response**				
CR	1 (1.2)	1 (2.4)	0	
PR	21 (25.3)	9 (22.0)	12 (28.6)	
SD	35 (42.2)	21 (51.2)	14 (33.3)	
PD	26 (31.3)	10 (24.4)	16 (38.1)	
**ORR**	22 (26.5)	10 (24.4)	12 (28.6)	0.666
**DCR**	57 (68.7)	31 (75.6)	26 (61.9)	0.178

Data are presented as number (percentage) of patients

CR, complete response; PR, partial response; SD, stable disease; PD, progressive disease; ORR objective response rate, (CR + PR) * 100 / total cases; DCR, disease control rate, (CR + PR + SD) * 100 / total cases, SMI^High^, high skeletal muscle mass; SMI^Low^, low skeletal muscle mass; UGC, unresectable gastric cancer

### Relationships of nutritional and inflammatory factors with SMI

Table 6 shows the relationships between various nutrition- and inflammation-based prognostic scores and SMI in patients with UGC. NLR was significantly higher in patients with SMI^Low^ than those with SMI^High^. (*p* = 0.047). However, there were no significant differences between the two groups regarding CRP, albumin, PNI, CAR, PLR, CEA, and CA19–9.

**Table 6:** Nutrition- and inflammation-based markers of patients with SMI^High^ and SMI^Low^ UGC

	SMI^High^ (n = 41)	SMI^Low^ (n = 42)	P value
CRP	0.789 ± 0.928	1.273 ± 2.731	0.245
Albumin	3.7 ± 0.5	3.5 ± 0.7	0.296
PNI	45.05 ± 5.73	42.4 ± 8.7	0.108
NLR	2.567 ± 1.081	4.110 ± 4.560	0.047
CAR	0.220 ± 0.255	0.514 ± 1.264	0.308
PLR	189.0 ± 103.7	224.9 ± 135.2	0.226
CEA	18.3 ± 49.1	16.7 ± 38.4	0.604
CA19–9	334.9 ± 1198.2	111.6 ± 416.2	0.610

Data are presented as the mean ± standard deviation or number (percentage) of patients

CA19–9, carbohydrate antigen; CEA, carcinoembryonic antigen; CRP, C-reactive protein; CAR, C-reactive protein-to-albumin ratio; NLR, neutrophil to lymphocyte ratio; PLR, platelet to lymphocyte ratio; PNI, the prognostic nutritional index; SMI^High^, high skeletal muscle mass; SMI^Low^, low skeletal muscle mass; UGC, unresectable gastric cancer

#### Univariate and multivariate analyses of patients with UGC

We performed univariate analysis of the clinicopathological factors considered prognostic for OS in patients with UGC. In the univariate analysis, ECOG PS, SMI, histological type, lines of chemotherapy (≧ 3rd line), and NLR were identified as prognostic indicators (Table 7). In the multivariate analysis, we included significant parameters that were identified in the univariate analysis. The multivariate analysis revealed that SMI, NLR, and lines of chemotherapy(≧ 3rd line) were independent prognostic factors (Table 7).

**Table 7:** Univariate and multivariate analyses of prognostic factors for OS in patients with UGC

	Univariate analysis			Multivariate analysis		
	Hazard ratio	95% CI	P value	Hazard ratio	95% CI	P value
Age (≧75 vs < 75)	1.148	0.680–1.938	0.605			
Gender (Female vs Male)	1.224	0.718–2.086	0.457			
ECOG PS (1,2 vs 0)	2.203	1.372–3.537	0.001	1.355	0.786–2.337	0.274
BMI (< 18.0 vs ≧18.0)	1.401	0.864–2.271	0.172			
SMI (Low vs High)	1.964	1.183–3.252	0.009	1.886	1.156–3.077	0.011
Histologic type (Differentiated vs Undifferentiated)	1.775	1.087–2.897	0.022	1.619	0.990–2.648	0.061
Peritoneum metastasis (Present vs Absent)	1.138	0.698–1.854	0.605			
Lymph node metastasis (Present vs Absent)	1.019	0.642–1.618	0.936			
Hematogenous metastasis (Present vs Absent)	1.101	0.675–1.793	0.702			
Lines of chemotherapy (≧2nd line vs < 2nd line)	0.958	0.513–1.789	0.893			
Lines of chemotherapy (≧3rd line vs <3rd line)	0.408	0.253–0.659	< 0.001	0.420	0.248–0.712	< 0.001
PNI (< 43.9 vs ≧43.9)	1.589	0.991–2.813	0.052			
NLR (≧2.881 vs < 2.881)	2.071	1.297–3.307	0.002	2.167	1.325–3.544	0.002
CAR (≧0.077 vs < 0.077)	1.361	0.854–2.171	0.195			
PLR (≧267 vs < 267)	1.490	0.885–2.510	0.134			

BMI, body mass index; CAR, C-reactive protein-to-albumin ratio; ECOG PS, Eastern Cooperative Oncology Group performance status; NLR, neutrophil to lymphocyte ratio; OS, overall survival; PLR, platelet to lymphocyte ratio; PNI, the prognostic nutritional index; SMI, skeletal musclemass; UGC, unresectable gastric cancer

## Discussion

In this study, the SMI^Low^ group had significantly more grade 3 or 4 side effects and were related to high NLR. The SMI^Low^ group had significantly less conversion to third-line chemotherapy than the SMI^High^ group and had a significantly worse prognosis than the SMI^High^ group.

In this study, the SMI^Low^ group had a significantly worse prognosis than the SMI^High^ group. Sarcopenia has been reported to indicate a poor prognosis in several cancers, including gastric cancer. Kamarajah et al. described that a meta-analysis of nine studies reporting OS after gastrectomy identified significantly worse survival in patients with preoperative sarcopenia [25]. However, there are few studies reporting the prognostic significance of sarcopenia in patients with UGC. Kouzu et al. retrospectively analyzed the prognostic significance of sarcopenia in 67 patients who experienced gastric cancer recurrence and found that sarcopenia was an independent negative prognostic factor [26]. This study had results similar to our results; however, the reason for poor prognosis with sarcopenia was unclear in patients with UGC. In patients with UGC, palliative chemotherapy was standard therapy to improve the survival rate. However, we often saw that once a clinical response had been achieved by chemotherapy, the effect might not be sustained, and the chemotherapy regimen had to be changed. It is important to use all available drugs to increase the survival rate of patients with UGC, and the importance of third-line treatment in gastric cancer has been reported [27, 28]. In this study, the number of patients who received third-line chemotherapy was significantly higher in the SMI^High^ group than in the SMI^Low^ group. One potential reason for poor prognosis in patients with low SMM might be the low rate of receiving third-line chemotherapy, as in the SMI^L^°^w^ group in our study.

In this study, the SMI^Low^ group had significantly more grade 3 or 4 side effects than the SMI^High^ group, and the result was similar to results reported by Kurk et al. [29] They retrospectively examined 414 patients with metastatic colorectal cancer treated with capecitabine-based chemotherapy and reported that sarcopenia and/or muscle loss was associated with an increased risk of dose-limiting toxicities. Likewise, Matsuura et al. reported that low SMM was associated with an increased risk of chemotherapy-induced toxicity [30]. They retrospectively examined 41 patients with gastric cancer undergoing neoadjuvant chemotherapy and revealed that low SMM was the only factor significantly associated with severe diarrhea in univariate and multivariate analyses. These results showed that low SMM was related to the high-grade toxicity of chemotherapy. However, the mechanism that associated low SMM with toxicity was unclear. This may be due to the clearance of 5-FU. 5-FU, the main drug for gastric cancer, is hydrophilic but widely distributed due to active transport [31]. This drug undergoes extensive metabolism primarily via dihydropyrimidine dehydrogenase [31], and variants of dihydropyrimidine dehydrogenase have been associated with an increased risk of 5-FU toxicity [32]. It is reported that patients with high SMM have increased 5-FU clearance [33]. These findings suggest that decreased clearance of 5-FU due to low SMM may be related to increased side effects. Another possible reason is that the dose of chemotherapy is highly dependent on the patient’s height and weight, and changes in body composition may not be taken into account [33, 34] . Patients with sarcopenia tend to receive more chemotherapeutic agents with relatively low lean body mass, and as a result, they are more likely to suffer toxicity. This suggests that there are still opportunities for improvement in current dosage calculation methods. There is also a need for research on the optimal adjustment method for sarcopenia when prescribing chemotherapeutic agents.

In this study, NLR was an independent prognostic factor, and it was significantly higher in patients with SMI^Low^ than those with SMI^High^. These results were similar to results reported by Feliciano et al. [35] They retrospectively examined 2470 patients with colorectal cancer and found that NLR was associated with sarcopenia, and high NLR independently predicted poor OS. Kim et al. also reported that sarcopenia was associated with higher NLR in 186 patients with small cell lung cancer [36]. These results suggested that high NLR was related to poor survival. High NLR reflected a decreased peripheral lymphocyte count or an elevated peripheral neutrophil count. Low preoperative lymphocyte count is reportedly related to poor survival in several types of cancer [37, 38]. Neutrophils are important components of several inflammatory responses, including interleukin-6 (IL-6) [39], which has dual tumor development and metastasis roles. A high neutrophil count is related to a poor prognosis [40]. Sarcopenia is associated with cytokines, including IL-6 [41]. These findings suggest that high NLR is related to poor prognosis and indicate that there may be a link between NLR and sarcopenia.

This study has several limitations. First, we have conducted this retrospective study with patients from a single institution, and the number of patients is not large. Second, the first-line chemotherapy is chosen based on 5-FU, but this was not consistent. This is a long-term study and the guidelines have changed over time. Chemotherapy is administered per the guidelines in place at the time of treatment. Third, the optimal cutoff SMI value has not been determined in patients with UGC. Fourth, we have enrolled patients with both recurrent gastric cancer and advanced gastric cancer. Fifth, information regarding inflammatory cytokines such as IL-6 is not available because of the retrospective design. Therefore, well-designed, randomized, prospective studies with larger populations are needed to confirm these findings.

In conclusion, patients with UGC with low SMI have significantly more grade 3 or 4 side effects than those with high SMI, and SMI is a useful prognostic marker of UGC. In patients with UGC with low SMI, the side effects of chemotherapy, particularly those related to gastrointestinal toxicity, should be carefully managed in subsequent treatment.

## Data Availability

The datasets used and analyzed during the current study are available from the corresponding author on reasonable request.

## Abbreviations

BMI: body mass index
CA19–9: carbohydrate antigen
CEA: carcinoembryonic antigen
CRP: C-reactive protein
CAR: C-reactive protein-to-albumin ratio
ECOG PS: eastern cooperative oncology group performance status
FN: febrile neutropenia
NLR: neutrophil-to-lymphocyte ratio
OS: overall survival
PLR: platelet-to-lymphocyte ratio
PNI: the prognostic nutritional index
SMI: skeletal muscle mass index
SMI^High^: high skeletal muscle mass
SMI^Low^: low skeletal muscle mass
SMM: skeletal muscle mass
UGC: Unresectable gastric cancer
CT: Computed tomography
MST: Median survival time
